# Mould-like Deterioration of Anji Salamander (*Hynobius amjiensis*) Egg Capsules: Adverse Effects, Potential Pathogenic Factors, and Conservation Intervention

**DOI:** 10.3390/jof12100747

**Published:** 2026-10-05

**Authors:** Haiyan Dong, Junzhe Qian, Xianting Wang, Zhedong Lang, Xiaolu Jin, Xu Zhou, Yuyang Dai, Wenjing Wang, Yichen Ping

**Affiliations:** 1 Department of Basic Medical Science, Huzhou Normal University, 759 Erhuan East Road, Huzhou 313000, China; tb6_my641jouh@dingtalk.com (X.J.); 71k_hh2tq0tue@dingtalk.com (X.Z.); 2023102327@stu.zjhu.edu.cn (Y.D.); 2023102328@stu.zjhu.edu.cn (W.W.); d9f-zge8lltjz@dingtalk.com (Y.P.); 2 Anji Salamander (*Hynobius amjiensis*) National Nature Reserve Management Center, No. 77-83, Building 1, Yongfu Commercial Street, Baofu Town, Anji County, Huzhou 313000, China; wangxianting2024@126.com (X.W.); 15257222607@163.com (Z.L.); 3 National-Local Joint Engineering Laboratory of Aquatic Animal Genetic Breeding and Nutrition (Zhejiang), Key Laboratory of Aquatic Animal Genetic Breeding and Nutrition of Chinese Academy of Fishery Sciences, Huzhou University, 759 Erhuan East Road, Huzhou 313000, China; 2024389015@stu.zjhu.edu.cn

**Keywords:** *Hynobius amjiensis*, fungal infection, embryonic mortality, developmental abnormalities, conservation intervention

## Abstract

Fungal infection is a major cause of hatching failure in amphibian embryos. However, studies in Hynobiidae remain limited. Across four breeding seasons (10 November 2022–15 April 2026), we combined field monitoring of hatching rates and mould-affected egg proportions, lesion phenotyping, 18S and 16S rRNA gene sequencing, and a conservation intervention to characterise egg-capsule mould-like deterioration in the Anji salamander (*Hynobius amjiensis*), identify candidate pathogenic microorganisms, and evaluate the effect of the intervention on the survival rate of *Hynobius amjiensis*. The proportion of mould-affected eggs differed significantly among breeding seasons (2.05%, 4.02%, 8.24%, and 7.09%; *p* < 0.001), coinciding with reduced rainfall and higher temperatures during the same periods. White hypha-like growth accompanied embryonic necrosis in affected egg capsules; a few hatchlings showed developmental abnormalities. Eukaryotic and bacterial community compositions differed significantly among sample groups (18S: *p* = 0.010; 16S: *p* = 0.045). The relative abundances of Ascomycota, Zoopagomycota, and Basidiomycota were higher in affected embryos, and those of Chytridiomycota and Mucoromycota were higher in affected intestinal samples, but no bacterial taxon was consistently highly abundant. The intervention group had a significantly higher hatching rate (65.47% vs. 46.57%; *p* < 0.001) and a significantly lower malformation rate (5.48% vs. 12.66%; *p* < 0.001) than the natural-condition group. These findings can inform disease prevention and control in hynobiid salamanders and other amphibians.

## 1. Introduction

Amphibian populations continue to decline worldwide, driven primarily by habitat loss, climate change, environmental pollution, invasive species, and infectious diseases [1]. Among these drivers, fungal diseases are important contributors to amphibian population declines. Chytridiomycosis, for example, has caused widespread population declines and mortality events [2,3]. Fungal disease can also occur during embryonic development in amphibians and reduce hatching rates [4,5,6]. Characterising lesion progression during embryonic development is therefore important for understanding amphibian early life history.

Embryonic survival and successful hatching directly affect the number of individuals entering subsequent life-history stages; disease during the embryonic stage can therefore affect reproductive recruitment in endangered populations [7,8,9]. In amphibians, egg capsules both limit direct contact between embryos and external microorganisms and provide an interface through which host-associated microorganisms can defend against environmental pathogens [10,11,12]. A previous study of the spotted salamander (*Ambystoma maculatum*) used 18S rRNA gene amplicon sequencing to characterise egg-capsule-associated eukaryotic communities, including algae, protists, and chytrid fungi [13]. In the same species, 16S rRNA gene amplicon sequencing was used to investigate bacterial diversity in egg-capsule fluid during embryonic development [11]. Similarly, a study of fire salamanders (*Salamandra salamandra*) used 16S rRNA gene sequencing to examine skin bacterial communities in relation to fungal disease [14]. Pathogen infection can cause localised embryonic mortality and spread to surrounding embryos [4,10,15]. Fungal growth and pathogenic processes are closely associated with environmental conditions, with temperature acting as an important regulator. The growth rate of water moulds on amphibian eggs can vary markedly with temperature [5], and chytrid infection and pathogenicity also show pronounced temperature dependence [16]. Air temperature and precipitation may alter the environmental stability of breeding pools. We found that excessive algal growth in some pools may lead to gas bubble disease in *Hynobius amjiensis* [17]. Temperature and rainfall during the breeding season therefore provide an important environmental context for the occurrence and progression of disease during amphibian embryogenesis.

*Hynobius amjiensis* is a critically endangered tailed amphibian endemic to China. It occurs mainly in high-elevation wetlands on Longwangshan, Anji, Zhejiang Province, and its wild population is small [18]. The species generally breeds from November to April of the following year and lays paired egg capsules in batches; fertilised eggs develop and hatch within the capsules. Embryos from different oviposition batches develop and hatch at different stages of the breeding season; earlier batches generally experience lower incubation temperatures and longer incubation periods [19,20,21,22]. Mould-like deterioration of *Hynobius amjiensis* egg capsules has long been observed, but in recent years breeding-season rainfall in its habitat has decreased and temperatures have increased [23,24], and egg-capsule mould-like deterioration has become more severe, leading to lower hatching rates [4,5,25]. Identifying the causes of this deterioration and implementing conservation interventions are therefore urgent. Studies of fish background choice have provided insights into environmental preferences and their potential applications in welfare-oriented husbandry [26,27]. Given the critically endangered status of *Hynobius amjiensis*, conservation interventions should balance potential conservation benefits with animal welfare by minimising unnecessary handling of egg capsules and disturbance to developing embryos.

The aim of this study was to characterise variation in egg-capsule mould-like deterioration across breeding seasons, assess its adverse effects, investigate associated microbial communities, and evaluate conservation intervention outcomes in *Hynobius amjiensis*. We hypothesised that mould-affected egg proportions would differ among breeding seasons, microbial community composition would differ among sample types, spreading deterioration would be associated with poorer hatching outcomes, and conservation intervention would be associated with improved hatching outcomes. Across four breeding seasons, we analysed egg numbers per egg-capsule pair, the proportion of mould-affected eggs, egg-capsule mould-like deterioration, embryonic mortality, and larval developmental abnormalities in *Hynobius amjiensis*, together with the corresponding meteorological data. We then used 18S and 16S rRNA gene amplicon sequencing to characterise eukaryotic (including fungal) and bacterial communities, respectively, in affected embryos and other host-associated samples and identify candidate taxa associated with mould-like deterioration. We also conducted a conservation intervention for affected egg capsules. This study may help assess the risks of fungal disease during embryonic development, guide prevention and control measures, and support targeted disease interventions.

## 2. Materials and Methods

### 2.1. Study Site and Study Subjects

Field monitoring was conducted from November 2022 to April 2026 in the Longwangshan National Nature Reserve for *Hynobius amjiensis*, Anji County, Zhejiang Province (30°28′ N, 119°42′ E). Monitoring spanned 10 November 2022 to 15 April 2023 (the 2022–2023 breeding season), 10 November 2023 to 15 April 2024 (the 2023–2024 breeding season), 10 November 2024 to 15 April 2025 (the 2024–2025 breeding season), and 10 November 2025 to 15 April 2026 (the 2025–2026 breeding season). Based on oviposition dates, egg capsules in each breeding season were divided into three batches: capsules laid from mid-to-late November of the previous year to the end of December were assigned to the first batch (B1); those laid from early January to mid-February of the current year were assigned to the second batch (B2); and those laid from mid-February to the end of March were assigned to the third batch (B3).

In each breeding season, we monitored 3 natural breeding pools and selected 3 egg-capsule pairs from each pool, for a total of 9 pairs. For each pair, we recorded the number of eggs, the number of mould-affected eggs, and the developmental status of the egg capsules and embryos. When the two individual egg capsules within a pair showed different lesion states, we recorded their lesion states and hatching outcomes separately.

### 2.2. Criteria for Identifying Egg Capsules and Larvae with Fungal Infection

**Figure 1:**
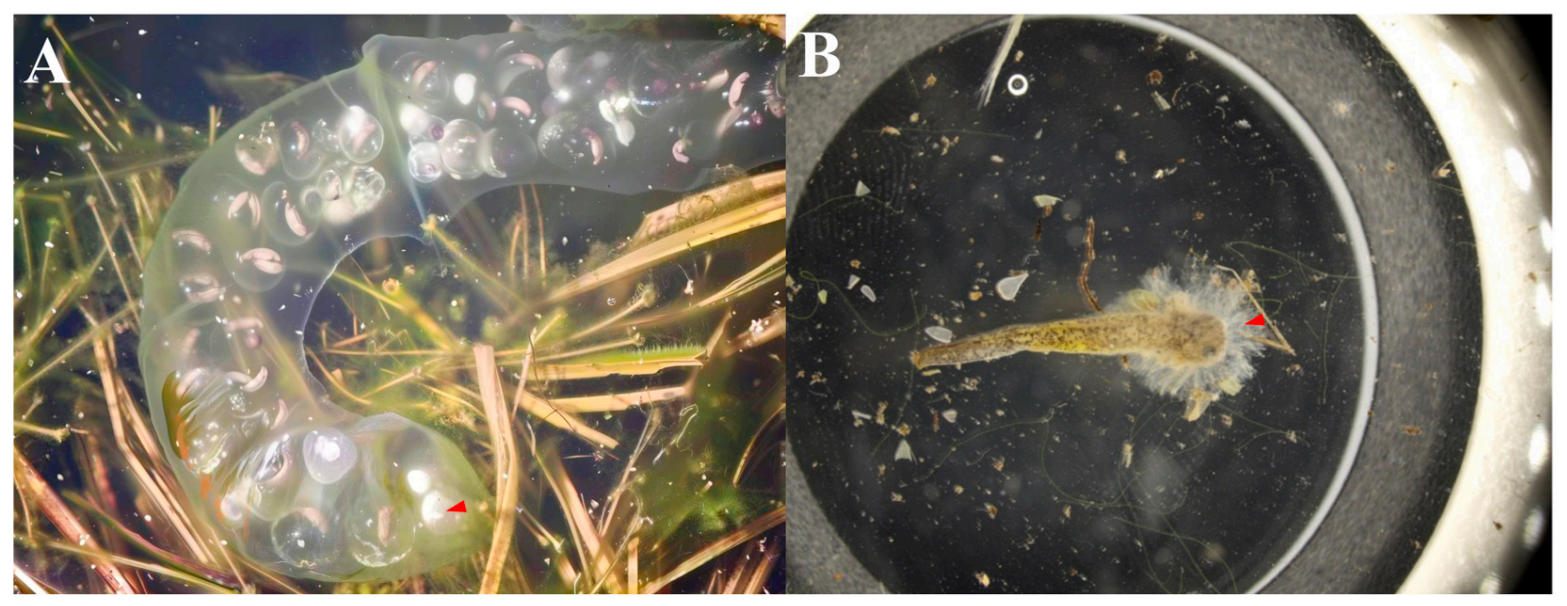
Morphological classification of phenotypes associated with mould-like deterioration in *Hynobius amjiensis* egg capsules and larvae. (**A**) Mould-like deterioration of eggs within an egg capsule, characterised by white hypha-like structures covering the egg surface and grey-white discolouration of the egg contents (red arrows). (**B**) Lesion phenotype in a larva, characterised by white hypha-like structures attached to or covering the body surface (red arrows).

Lesion categories were defined using visible morphological features. Eggs within a capsule were classified as showing spreading mould-like deterioration when white hypha-like growth was visible on their surfaces, their contents had turned grey-white, and the lesion was observed to spread to nearby eggs (Figure 1A). Unfertilised or naturally dead eggs that turned grey-white or lysed were not classified as showing spreading mould-like deterioration if no spread to neighbouring eggs was observed during serial observation. Larvae or subadults with white hypha-like material attached to their body surface were classified as infected (Figure 1B). These criteria were used to classify morphology and provisionally delineate lesion extent, but final determination of fungal infection also required sequencing results.

### 2.3. Proportion of Mould-Affected Eggs and Egg Hatching Rates in Infected Egg Capsules Across Oviposition Batches

In each breeding season, each egg-capsule pair was used as the observational unit. We recorded the total number of eggs and the number of mould-affected eggs, and calculated the proportion of mould-affected eggs as follows: proportion of mould-affected eggs (%) = number of mould-affected eggs/total number of eggs × 100%. Natural hatching outcomes among oviposition batches were assessed using each individual egg capsule as the observational unit. We recorded the initial number of eggs, the number of successfully hatched larvae and the number of malformed larvae, and calculated the hatching and malformation rates as follows: hatching rate (%) = number of successfully hatched larvae/initial total number of eggs × 100%; malformation rate (%) = number of malformed larvae after hatching/initial total number of eggs × 100%.

### 2.4. Sample Collection and Phenotype Observation

Samples for 18S rRNA community analysis comprised stomach samples (Stomach), intestine samples (Intestine), and affected embryo samples (Embryo), with 3 biological replicates per group (*n* = 3). Samples for 16S rRNA community analysis comprised faeces from healthy individuals (HF), mould-affected embryos (Embryo), and intestinal contents from deceased affected individuals (IC), with 3 biological replicates per group (*n* = 3). The 18S and 16S datasets were obtained from independent biological samples, and the sample types were not fully matched between the two datasets. Sample identifiers and sources are provided in Supplementary Table S1. All samples were collected using sterile instruments, snap-frozen in liquid nitrogen and stored at −80 °C. Egg capsules, eggs, and embryos were examined under a stereomicroscope (Carl Zeiss Microscopy GmbH, Jena, Germany). Larvae and subadults were imaged using either a stereomicroscope or a smartphone (Huawei Technologies Co., Ltd., Shenzhen, China), depending on body size; we recorded only visible morphological phenotypes.

### 2.5. Amplicon Sequencing and Bioinformatics Analysis

Samples were transported on dry ice to Majorbio Bio-Pharm Technology Co., Ltd. (Shanghai, China), where DNA extraction, PCR amplification, library construction, and paired-end sequencing (2 × 250 bp) on an Illumina NextSeq 2000 (Illumina, Inc., San Diego, CA, USA) were performed. The 18S rRNA and 16S rRNA genes were amplified using primer pairs SSU0817F/SSU1196R and 338F/806R, respectively. Details of the reaction mixtures, amplification programmes, product purification, library construction, and primer sequences are provided in Supplementary Method S1 and Supplementary Table S1.

After read merging, demultiplexing, quality filtering, and chimera removal, raw reads were clustered into operational taxonomic units (OTUs) at 97% similarity using UPARSE 7.0.1090 [28]. Representative OTU sequences were taxonomically annotated using RDP Classifier 2.11 with a bootstrap confidence threshold of 0.7 [29]; 16S rRNA gene sequences were taxonomically annotated against the SILVA 138 database [30] and cross-validated using the RDP 11.5 [31] and Greengenes 13.5 databases [32]. The 18S rRNA gene sequences were taxonomically annotated against the SILVA 138 SSUref database [30]. OTU abundance tables and taxonomic annotations are provided in Supplementary Tables S3–S6. Taxon relative abundances were Z-score standardised before heatmap construction. Circos plots were used to show the relative contributions of major taxa to sample groups. UpSet analysis was based on a group-level presence/absence matrix; a taxon was defined as present in a group when detected in at least 2 of the 3 biological replicates.

### 2.6. Conservation Intervention for Embryos and Hatching Observations

Natural hatching outcomes were compared between egg capsules with non-spreading and spreading mould-like deterioration across the three oviposition batches B1, B2, and B3. Additional egg capsules with spreading mould-like deterioration were included in the conservation intervention. The intervention experiment comprised a natural-condition group (N, *n* = 8) and a post-intervention low-temperature incubation group (L, *n* = 7). The N group was observed under natural conditions, whereas the L group was transferred to the laboratory, where affected eggs were removed before low-temperature incubation. For the intervention, mould-affected eggs were first aspirated using a 20 G, 100 mm puncture needle (Zhuji Pengtian Medical Instrument Co., Ltd., Zhuji, Zhejiang, China). The resulting cavity was rinsed three times for 30–60 s each with 0.001% povidone-iodine working solution using the same volume as that aspirated. The cavity was then rinsed three times with sterile Ringer’s solution containing 2 mg/L vitamin C to reduce residual povidone-iodine. After rinsing, the cavity was refilled with 0.8% sterile Ringer’s solution gel using the same volume as that aspirated. When an egg capsule was damaged, it was gently ligated with sterile cotton thread to maintain its local structure as far as possible. The treated egg capsules were placed in a glass tank (50 × 30 × 16 cm) until hatching; 3 days later, the larvae were separated and fed live prey. Water conditions were based on measurements from natural breeding pools in the reference habitat. Water temperature was maintained at 4–8 °C before hatching and at 8–15 °C after hatching (dissolved oxygen, 7.700–7.900 mg/L; pH, 6.9–7.0; phosphate, 0.045 ± 0.005 mg/L; zinc, 0.05 ± 0.01 mg/L). A full-spectrum 12-h light-dark cycle was used, and disturbance was minimised throughout incubation. Detailed reagent formulations and treatment procedures are provided in Supplementary Method S2.

### 2.7. Meteorological Data

Monthly mean air temperature and monthly precipitation during the study were obtained from records of the Anji County meteorological station of the China Meteorological Administration (station ID: 58446) to describe the regional climatic background across the four breeding seasons. Monthly mean air temperature was corrected using the vertical temperature lapse rate [33]

T_H_ = T_S_ − 0.006 × ΔH

In the equation, *T*_H_ is the approximate monthly mean air temperature at the breeding site, *T*_S_ is the monthly mean air temperature at the meteorological station, and *ΔH* is the elevation difference between the breeding site and the meteorological station (1300 m). The corrected temperatures were used only to describe the approximate regional temperature background of the breeding site and do not represent actual water temperatures in natural breeding pools. Because precipitation in mountainous areas is spatially heterogeneous, monthly precipitation was not corrected for elevation. Cumulative precipitation for each breeding season was calculated as the sum of monthly precipitation from November of the current year to March of the following year. Methods for obtaining, correcting, and summarising the meteorological data are provided in Supplementary Method S3 and Supplementary Table S2.

### 2.8. Statistical Analysis

The number of eggs per egg-capsule pair was analysed using a linear model (LM), with breeding season and breeding pool as fixed effects. Normality of model residuals was assessed using the Shapiro–Wilk test, and homogeneity of variance using Levene’s test. After a significant overall effect of breeding season was detected, pairwise comparisons of estimated marginal means between breeding seasons were conducted with Tukey adjustment for multiple comparisons. The proportion of mould-affected eggs was analysed using a binomial generalised linear model (GLM), with breeding season and breeding pool as fixed effects. After a significant overall effect of breeding season was detected, post hoc pairwise comparisons between breeding seasons were conducted with Tukey adjustment for multiple comparisons. Natural hatching rates among the B1, B2, and B3 oviposition batches were analysed using the Kruskal–Wallis *H* test; after a significant overall difference was detected, Dunn’s test with Bonferroni correction was applied. Hatching rates and malformation rates in the natural-condition group and post-intervention low-temperature incubation group were analysed using two-sided Mann–Whitney *U* tests. Community structure was analysed separately for the 18S and 16S rRNA datasets. Bray–Curtis dissimilarities were calculated from relative-abundance data, and principal coordinates analysis (PCoA) was used to visualise differences in community composition among samples. Differences in community composition among groups were tested using PERMANOVA (Adonis) [34]. We used STAMP (https://beikolab.cs.dal.ca/software/STAMP, accessed on 2 October 2026) for pairwise differential-abundance analyses between groups in both the 18S and 16S rRNA datasets [35]. Group comparisons used two-sided *t*-tests. *p* values were adjusted for multiple testing using the Benjamini–Hochberg method [36], with adjusted *p* < 0.05 considered statistically significant. Except for analyses adjusted for multiple comparisons, all tests were two-sided, with the significance level set at *p* < 0.05. Statistical analyses were performed in SPSS 25.0, and figures were prepared using GraphPad Prism 10.0 and OriginPro 2026.

## 3. Results

### 3.1. Egg Number and the Proportion of Mould-Affected Eggs Differed Among the Four Breeding Seasons

**Figure 2:**
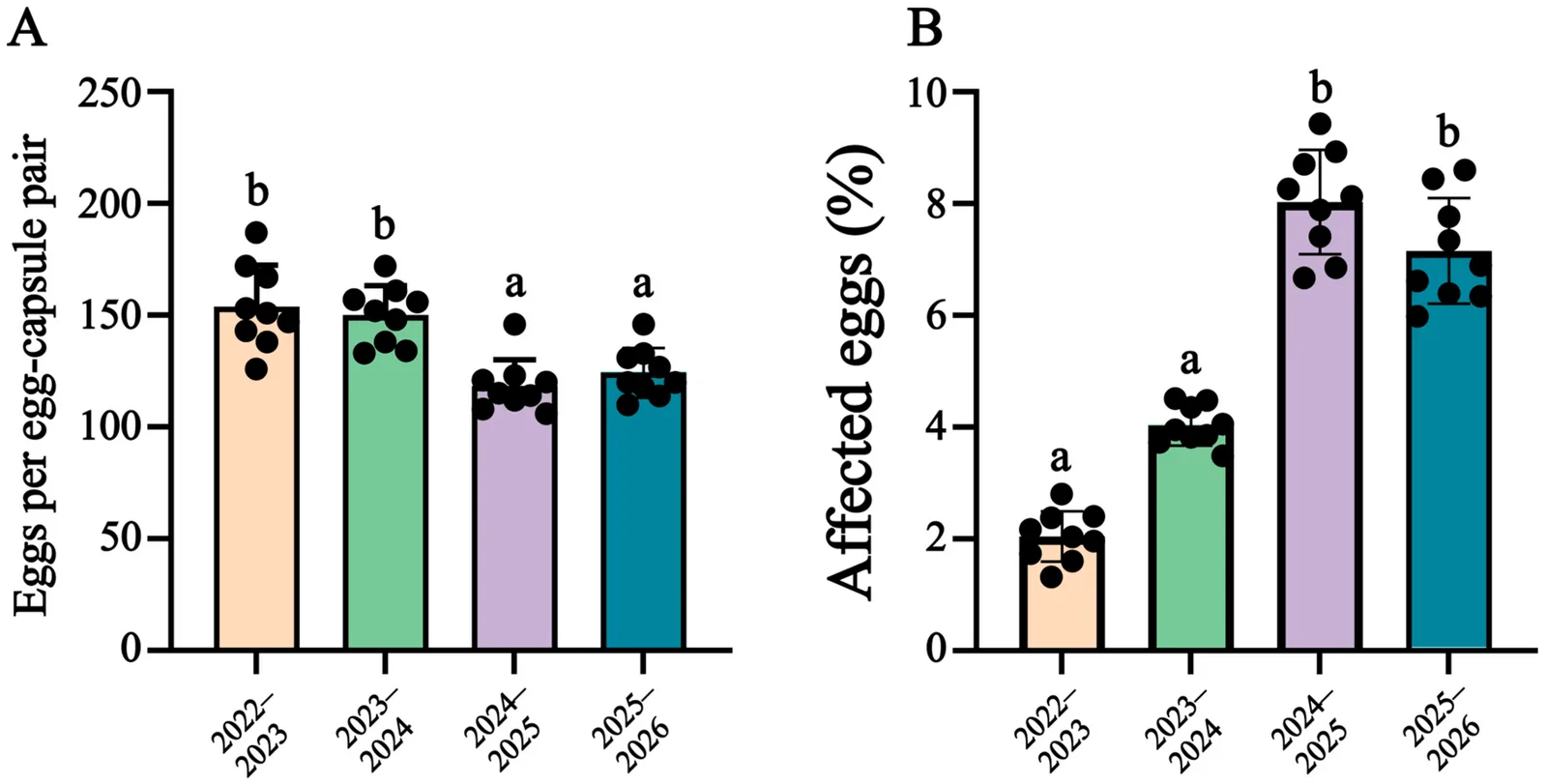
Egg numbers and proportions of mould-affected eggs in *Hynobius amjiensis* egg capsules across four breeding seasons from 2022 to 2026. (**A**) Number of eggs per egg-capsule pair in each breeding season. (**B**) Proportion of mould-affected eggs per egg-capsule pair across the four breeding seasons. Each breeding season included 9 egg-capsule pairs from 3 fixed natural breeding pools (3 pairs per pool); each point represents 1 egg-capsule pair, and the bars and error bars show mean ± SD. Differences among groups were tested using a linear model (**A**) and a binomial generalised linear model (**B**), followed by Tukey-adjusted pairwise comparisons. Different lowercase letters indicate significant differences among groups (*p* < 0.05).

In the four breeding seasons, the numbers of eggs per egg-capsule pair were 153.78 ± 18.71, 150.11 ± 13.17, 118.33 ± 11.86, and 123.33 ± 11.10, respectively (Figure 2A). Egg number per egg-capsule pair differed significantly among the four breeding seasons (*F*_3,30_ = 11.46, *p* < 0.001). Egg numbers in 2024–2025 and 2025–2026 were lower than those in 2022–2023 and 2023–2024 (Tukey, all *p* < 0.001; Figure 2A). The proportions of mould-affected eggs in the four breeding seasons were 2.05 ± 0.45%, 4.02 ± 0.35%, 8.24 ± 0.98%, and 7.09 ± 0.95%, respectively (Figure 2B). The proportion of mould-affected eggs differed significantly among the four breeding seasons (Wald χ^2^_3_ = 49.30, *p* < 0.001). The proportions of mould-affected eggs in 2022–2023 and 2023–2024 were lower than those in 2024–2025 and 2025–2026 (Tukey, all *p* < 0.001; Figure 2B). Cumulative precipitation during the 2022–2023, 2023–2024, 2024–2025, and 2025–2026 breeding seasons was 317.0, 390.1, 255.9, and 239.2 mm, respectively. Changes in monthly mean air temperature over the same periods are shown in Supplementary Figure S1. The proportion of mould-affected eggs was highest in 2024–2025, whereas cumulative precipitation was highest in 2023–2024 and lowest in 2025–2026.

### 3.2. Affected Egg Capsules and Post-Hatching Individuals Showed Hypha-like Growth and Structural Damage

**Figure 3:**
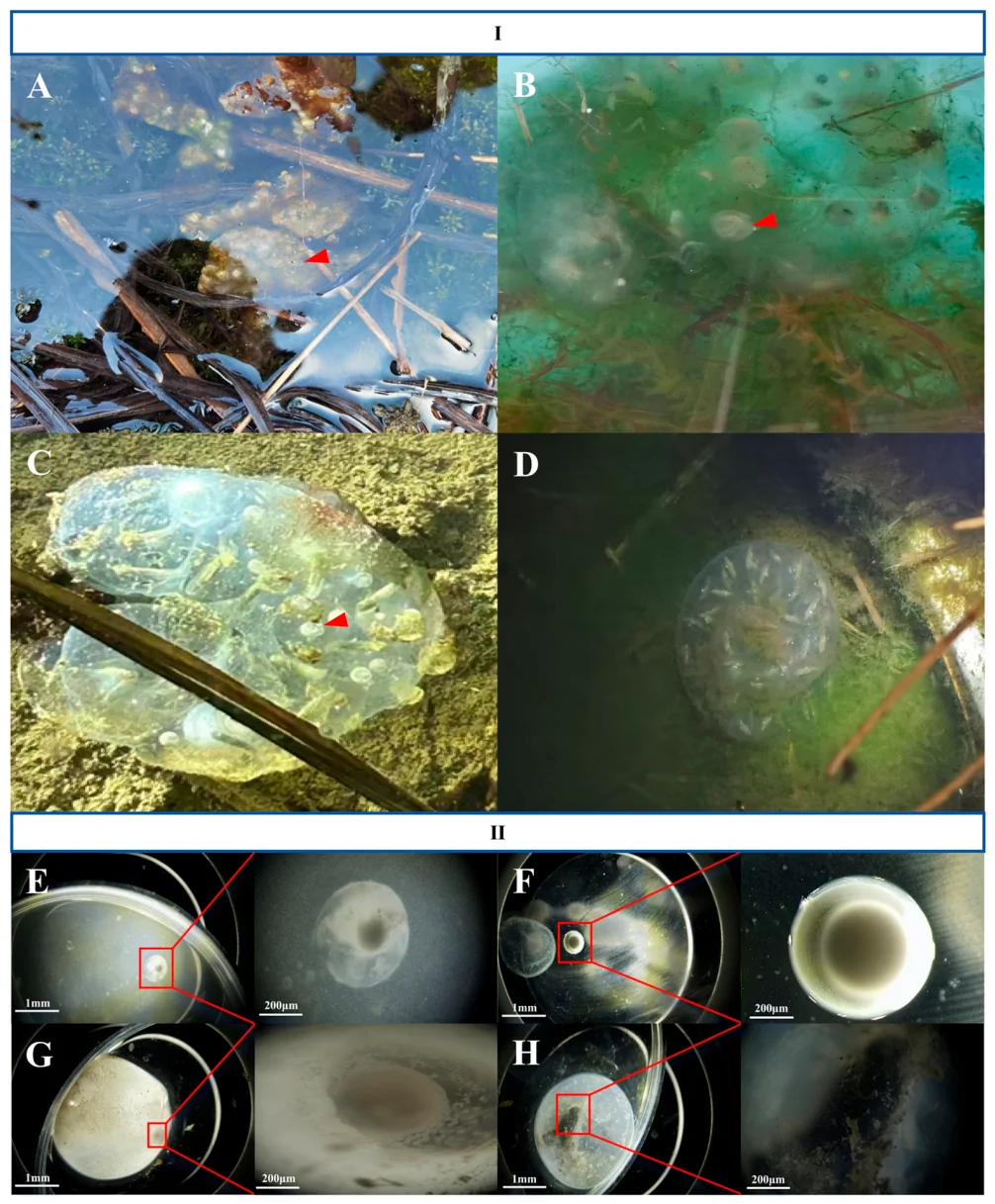
Macroscopic and microscopic phenotypes of affected *Hynobius amjiensis* egg capsules and embryos. (**I**) Macroscopic phenotypes of affected egg capsules (**A**–**D**); (**II**) microscopic phenotypes of affected eggs and embryos (**E**–**H**). Red arrows indicate representative areas, and red boxes indicate magnified regions. (**A**) An egg capsule with a relatively intact membrane and white hypha-like growth inside. (**B**) Membrane rupture accompanied by the dispersal of eggs. (**C**) Transparent developing embryos and grey-white eggs within the same egg capsule. (**D**) An egg capsule without apparent lesions. (**E**) Hypha-like growth around an egg. (**F**) Separation of the egg membrane from the internal structures and grey-white discolouration of the egg contents. (**G**) Disintegration of the internal egg structure. (**H**) Hypha-like growth in contact with the embryo surface, accompanied by embryonic necrosis.

**Figure 4:**
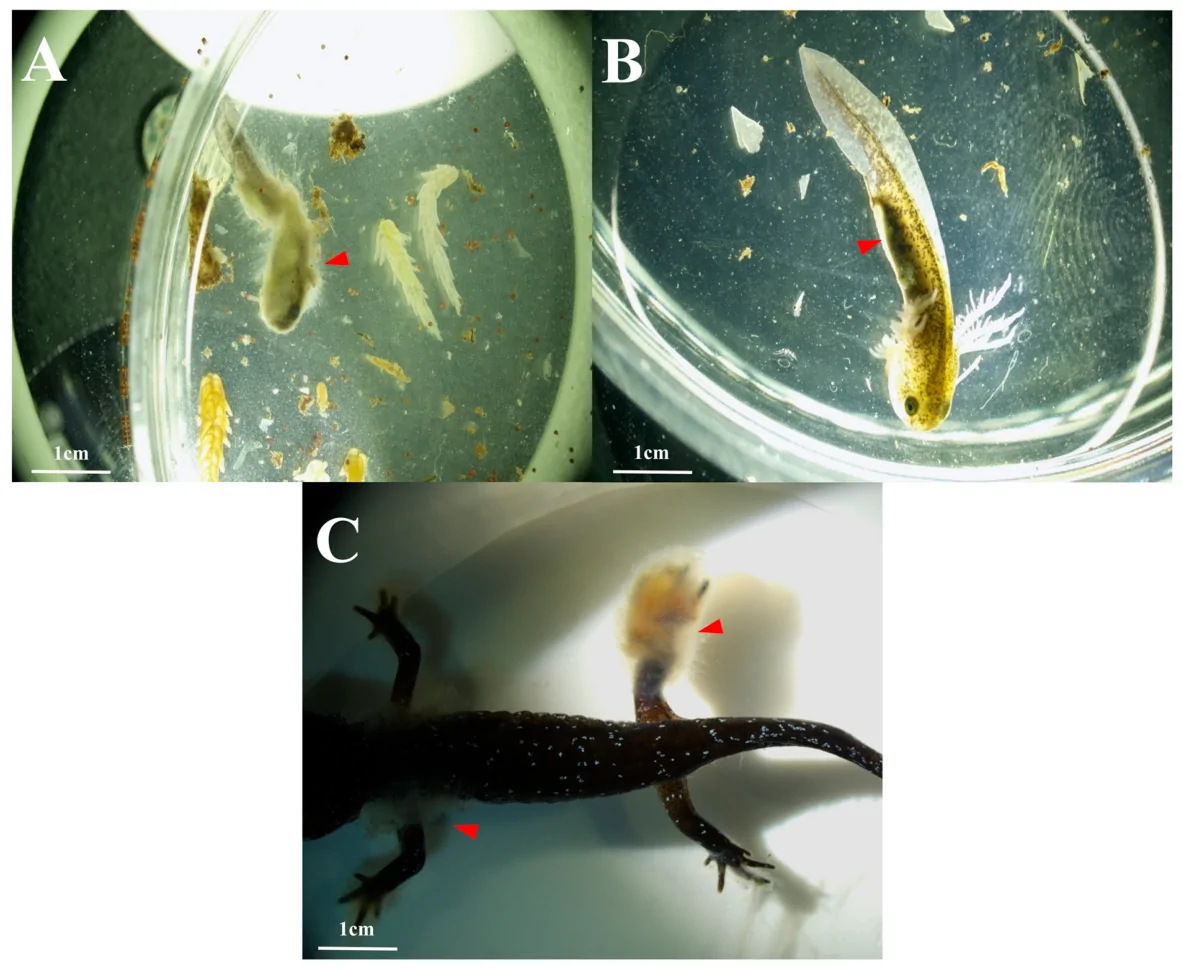
Visible lesion characteristics in *Hynobius amjiensis* larvae and subadults. Red arrows indicate representative observation areas. (**A**) A larva with white hypha-like structures covering the body surface. (**B**) A larva with localised abdominal swelling or bulging. (**C**) A subadult with white hypha-like structures attached to a wounded area.

In egg capsules whose membranes remained intact, white hypha-like growth was visible around some eggs (Figure 3A). In other affected egg capsules, the membrane had ruptured and the eggs were dispersed (Figure 3B). Some affected egg capsules contained both transparent developing embryos and grey-white eggs (Figure 3C). Egg capsules without apparent lesions were transparent and gelatinous, with clearly visible internal embryonic structures (Figure 3D). Microscopy showed hypha-like growth around affected eggs (Figure 3E). As the lesions progressed, the egg membrane separated from the internal structures, the egg contents turned grey-white, and the internal structure disintegrated (Figure 3F,G). In some samples, hypha-like growth contacted the embryo surface and was accompanied by embryonic necrosis (Figure 3H). Similar hypha-like structures were also visible on some post-hatching individuals. Affected larvae showed white hypha-like structures covering the body surface (Figure 4A) or localised swelling (Figure 4B); in some subadults, white hypha-like structures adhered to wounded areas (Figure 4C).

### 3.3. Larval Developmental Abnormalities in Individuals from Affected Egg Capsules

**Figure 5:**
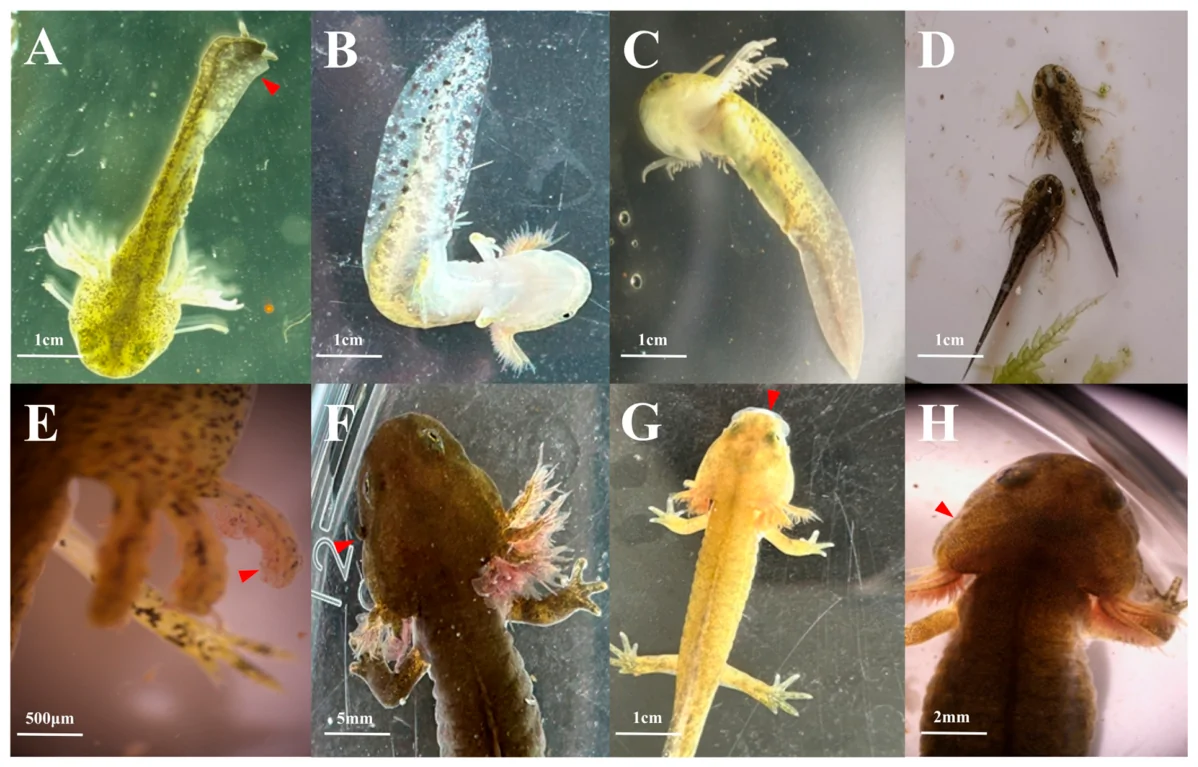
Representative developmental abnormalities in surviving larvae from affected egg capsules and normal reference morphology. Red arrows indicate representative observation areas. (**A**) A wrinkled tail structure. (**B**,**C**) Abnormal curvature of the body axis. (**D**) External morphology of a normally developing individual. (**E**) Thickened, shortened, or irregular external gill filaments. (**F**) A nodular structure at the site where the balancer had detached. (**G**) A persistently open snout with a fish-mouth-like appearance. (**H**) Local morphology at the balancer-detachment site in a normal individual.

Multiple external morphological abnormalities were observed in larvae hatched from some affected egg capsules: a wrinkled tail structure (Figure 5A); L- or C-shaped curvature of the body axis (Figure 5B,C); thickened, shortened, or irregular external gill filaments (Figure 5E); a fleshy, nodule-like protrusion at the site where the balancer had detached (Figure 5F); and a persistently open snout with a fish-mouth-like appearance (Figure 5G). By contrast, normally developing individuals had a relatively straight body axis and intact external structures (Figure 5D). Only a slight depression or evidence of healing was visible at the site where the balancer had detached (Figure 5H).

### 3.4. Eukaryotic and Bacterial Community Composition Differed Among Sample Groups

**Figure 6:**
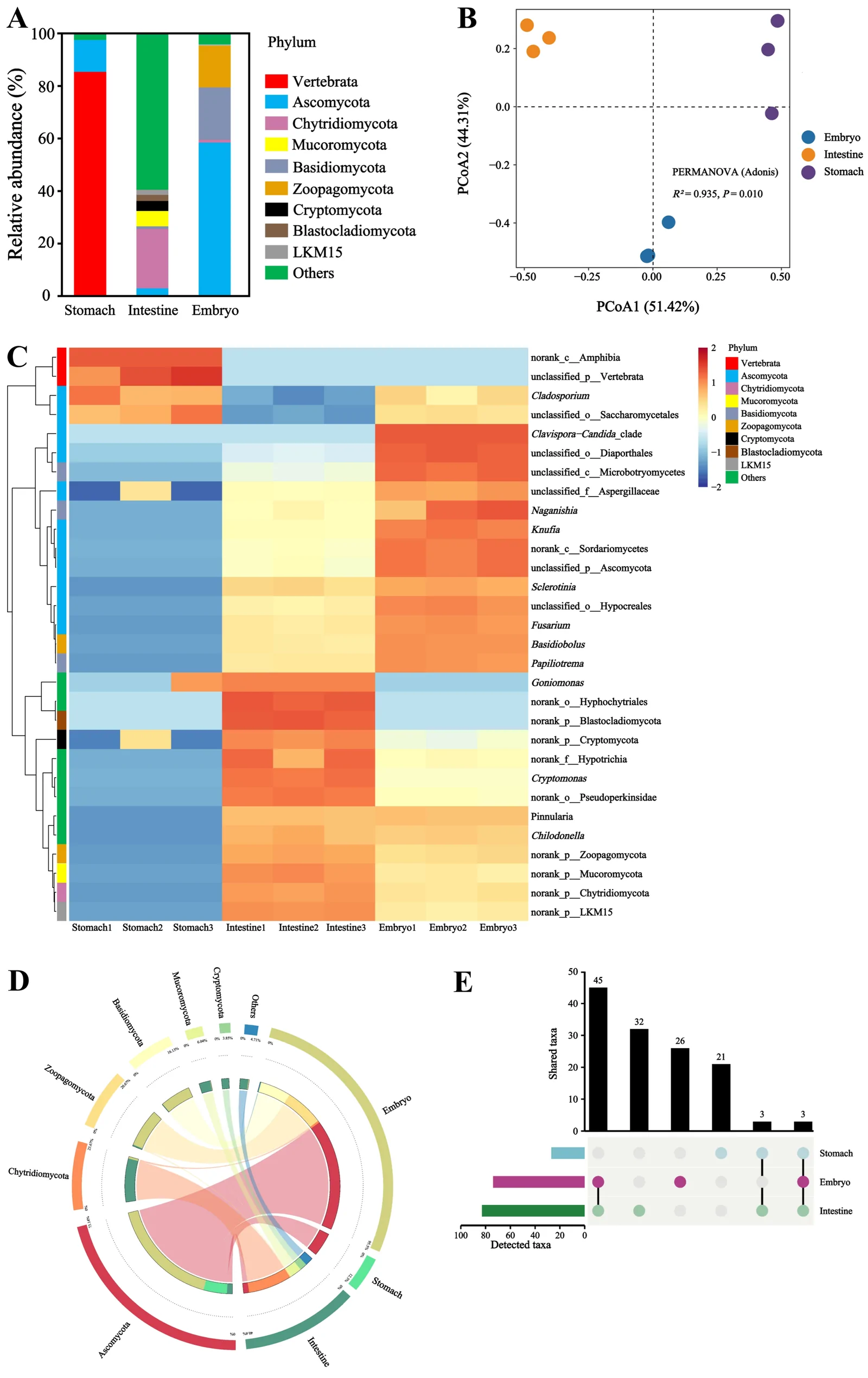
Eukaryotic community characteristics and fungal taxon distributions in *Hynobius amjiensis* Stomach, Intestine, and Embryo samples based on 18S rRNA gene amplicon sequencing. (**A**) Mean relative-abundance composition of the major eukaryotic taxa at the phylum level in Stomach, Intestine, and Embryo samples; taxa not shown separately were grouped as “Others”. (**B**) Principal coordinates analysis (PCoA) based on Bray–Curtis dissimilarities, showing differences in community composition among Stomach, Intestine, and Embryo samples. Each point represents 1 independent biological sample, and different colours denote different sample groups; differences in community composition among groups were tested using PERMANOVA (Adonis) (*R*^2^ = 0.935, *p* = 0.010). (**C**) Hierarchical-clustering heatmap of the relative abundances of the major eukaryotic taxa in 9 samples. Relative abundances were Z-score standardised; colours from blue to red indicate standardised abundance from low to high, and the annotation colour bar on the left indicates the phylum to which each taxon belongs. (**D**) Circos plot showing the relative distributions of the major fungal phyla across the Stomach, Intestine, and Embryo groups; ribbon width indicates the relative contribution of each fungal phylum to each sample group. (**E**) UpSet plot showing shared and group-specific taxa among Stomach, Intestine, and Embryo samples. A taxon was defined as present in a group when, among the 3 biological replicates in that group, it was detected in at least 2 samples; the upper bar chart shows the number of taxa in each intersection, the horizontal bar chart on the left shows the total number of taxa detected in each group, and the connected dots below indicate the corresponding intersections among groups. Each group comprised 3 independent biological samples (*n* = 3).

**Figure 7:**
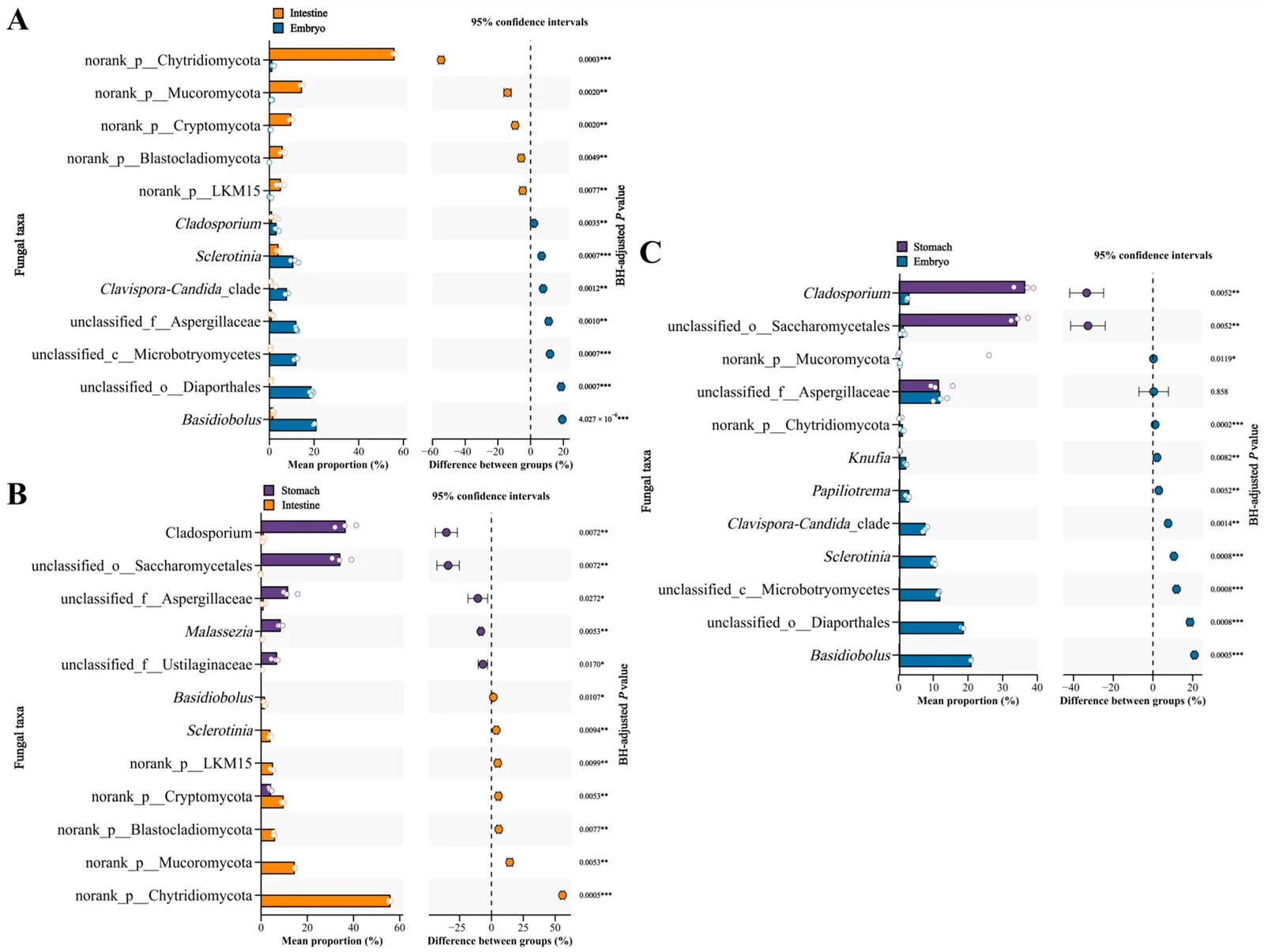
Pairwise differential-abundance analysis of fungal taxa in *Hynobius amjiensis* Stomach, Intestine and Embryo samples. STAMP was used for pairwise comparisons of fungal taxa among Stomach, Intestine, and Embryo samples: (**A**) Intestine versus Embryo; (**B**) Stomach versus Intestine; (**C**) Stomach versus Embryo. The left side of each panel shows the mean relative abundance of the corresponding taxa in the two sample groups and the values for each independent biological sample; the right side shows the difference between the two mean relative abundances and its 95% confidence interval. Differences between groups were tested using two-sided *t*-tests, and *p* values were adjusted for multiple testing using the Benjamini–Hochberg (BH) method. * *p* < 0.05, ** *p* < 0.01 and *** *p* < 0.001. Each group comprised 3 independent biological samples (*n* = 3).

18S rRNA gene amplicon analysis showed that eukaryotic community composition differed among the Stomach, Intestine, and Embryo groups (Figure 6). At the phylum level, Stomach samples were composed mainly of Vertebrata sequences; the relative abundances of Chytridiomycota, Mucoromycota, and Cryptomycota were higher in Intestine samples; and Ascomycota was the predominant fungal group in Embryo samples, in which Basidiomycota and Zoopagomycota were also detected (Figure 6A). PCoA showed separation among the three sample groups, and community composition differed significantly among them (PERMANOVA: *R*^2^ = 0.935, *p* = 0.010; Figure 6B). STAMP pairwise comparisons further showed that the relative abundances of taxa within Chytridiomycota, Mucoromycota, and Cryptomycota were higher in Intestine than in Embryo samples, whereas the relative abundance of *Basidiobolus* was higher in Embryo samples. The relative abundances of taxa within *Cladosporium* and Saccharomycetales were higher in Stomach than in both Intestine and Embryo samples (BH-adjusted *p* < 0.05; Figure 7).

**Figure 8:**
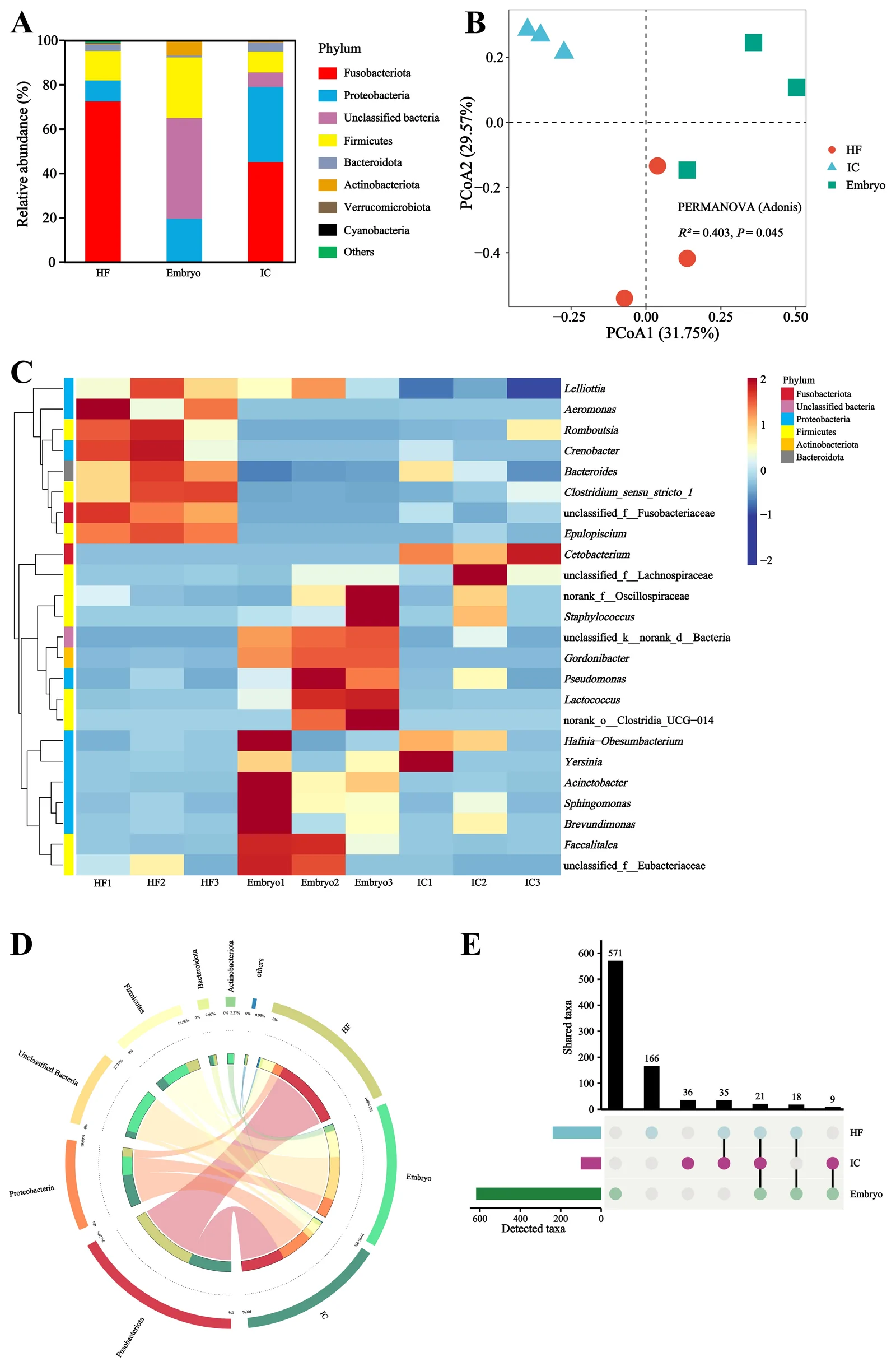
Bacterial community characteristics of faeces from healthy *Hynobius amjiensis* individuals, affected embryos, and intestinal-content samples based on 16S rRNA gene amplicon sequencing. (**A**) Mean relative-abundance composition of the major bacterial taxa at the phylum level in samples of faeces from healthy individuals (HF), affected embryo samples (Embryo), and intestinal-content samples (IC); taxa not shown separately were grouped as “Others”. (**B**) Principal coordinates analysis (PCoA) based on Bray–Curtis dissimilarities, showing differences in community composition among HF, Embryo, and IC samples. Each point represents 1 independent biological sample, and different colours and shapes denote different sample groups; differences in community composition among groups were tested using PERMANOVA (Adonis) (*R*^2^ = 0.403, *p* = 0.045). (**C**) Hierarchical-clustering heatmap of the relative abundances of the major bacterial taxa in 9 samples. Relative abundances were Z-score standardised; colours from blue to red indicate standardised abundance from low to high, and the annotation colour bar on the left indicates the phylum to which each taxon belongs. (**D**) Circos plot showing the relative distributions of the major bacterial phyla across the HF, Embryo, and IC groups; ribbon width indicates the relative contribution of each bacterial phylum to each sample group. (**E**) UpSet plot showing shared and group-specific taxa among HF, Embryo, and IC samples. A taxon was defined as present in a group when, among the 3 biological replicates in that group, it was detected in at least 2 samples; the upper bar chart shows the number of taxa in each intersection, the horizontal bar chart on the left shows the total number of taxa detected in each group, and the connected dots below indicate the corresponding intersections among groups. Each group comprised 3 independent biological samples (*n* = 3).

**Figure 9:**
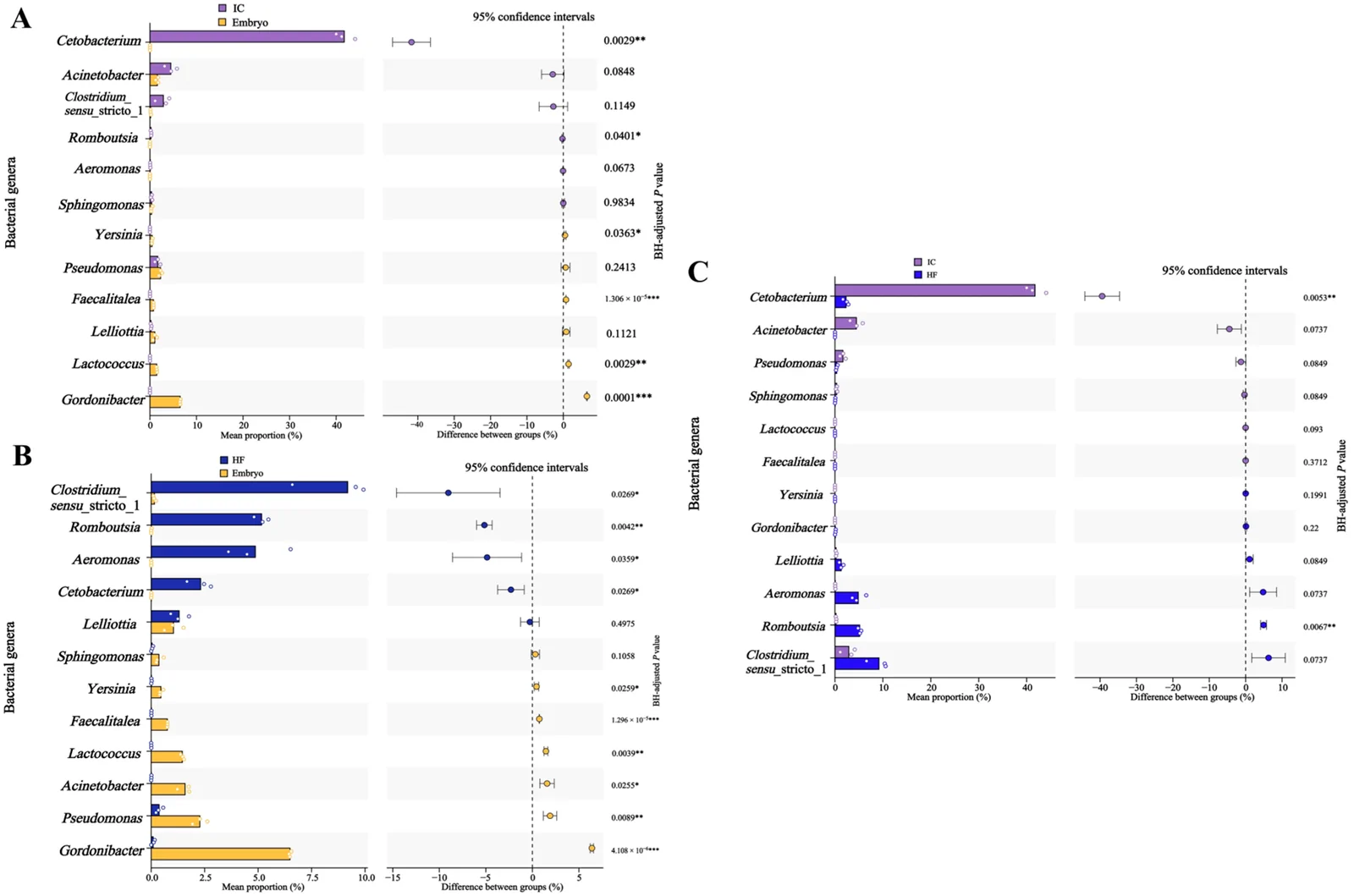
Pairwise differential-abundance analysis of bacterial taxa in HF, Embryo, and IC samples. STAMP was used for pairwise comparisons of bacterial taxa among HF, Embryo, and IC samples: (**A**) Embryo versus IC; (**B**) HF versus Embryo; (**C**) HF versus IC. The left side of each panel shows the mean relative abundance of the corresponding taxa in the two sample groups and the values for each independent biological sample; the right side shows the difference between the two mean relative abundances and its 95% confidence interval. Differences between groups were tested using two-sided *t*-tests, and *p* values were adjusted for multiple testing using the Benjamini–Hochberg (BH) method. * *p* < 0.05, ** *p* < 0.01 and *** *p* < 0.001. Each group comprised 3 independent biological samples (*n* = 3).

16S rRNA gene amplicon analysis showed that bacterial community composition differed among the HF, Embryo, and IC groups (Figure 8). At the phylum level, HF samples were dominated by Fusobacteriota, Firmicutes had a higher relative abundance in Embryo samples, and IC samples were composed mainly of Fusobacteriota and Proteobacteria (Figure 8A). PCoA showed separation of the three sample groups in ordination space and a significant difference in community composition (PERMANOVA: *R*^2^ = 0.403, *p* = 0.045; Figure 8B). STAMP pairwise comparisons showed that the relative abundances of *Yersinia*, *Faecalitalea*, *Lactococcus*, and Gordonibacter were higher in Embryo than in both HF and IC, the relative abundance of *Cetobacterium* was higher in IC than in HF and Embryo, and the relative abundance of *Romboutsia* was higher in HF than in IC and Embryo (BH-adjusted *p* < 0.05; Figure 9).

### 3.5. Natural Hatching and Conservation-Intervention Outcomes for Non-Spreading and Spreading Mould-like Deterioration

**Figure 10:**
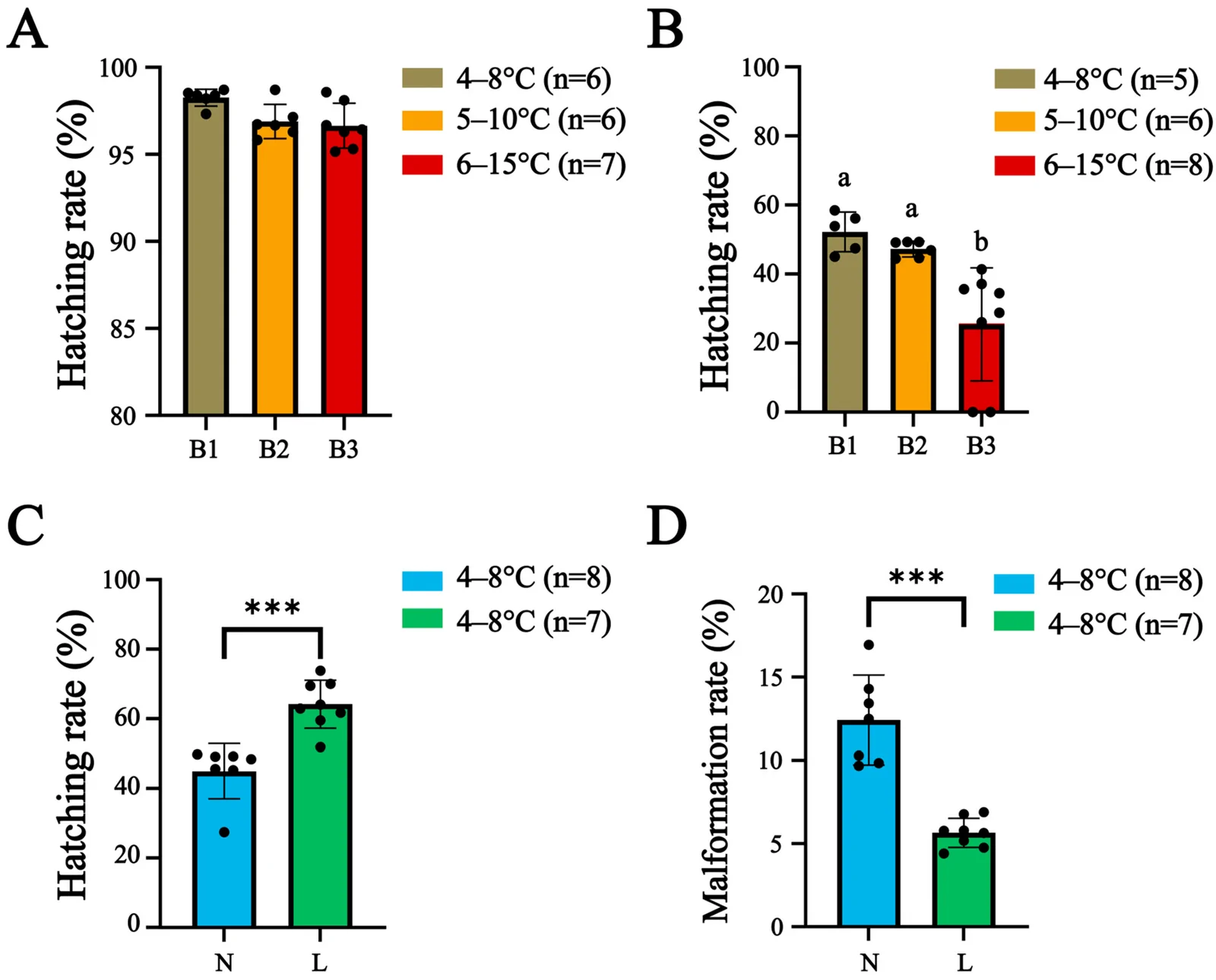
Natural hatching outcomes and effects of conservation intervention for *Hynobius amjiensis* egg capsules from different oviposition batches. (**A**) Natural hatching rates of egg capsules with non-spreading mould-like deterioration in oviposition batches B1, B2, and B3, with sample sizes of *n* = 6, 6, and 7, respectively. (**B**) Natural hatching rates of egg capsules with spreading mould-like deterioration in the three oviposition batches, with sample sizes of *n* = 5, 6, and 8, respectively. Overall comparisons in (**A**,**B**) used Kruskal–Wallis tests, followed by Dunn tests with Bonferroni adjustment for post hoc pairwise comparisons; different lowercase letters in (**B**) indicate significant adjusted differences among groups (*p* < 0.05). (**C**) Hatching rates in the natural-condition group (N, *n* = 8) and the post-intervention low-temperature incubation group (L, *n* = 7) for egg capsules with spreading mould-like deterioration. (**D**) Malformation rates in the N and L groups, calculated using the initial number of eggs as the denominator. (**C**,**D**) were analysed using two-sided Mann–Whitney *U* tests. Bars and error bars show mean ± SD, and each point represents 1 independent egg capsule. *** *p* < 0.001.

The natural hatching rates of egg capsules with non-spreading mould-like deterioration in oviposition batches B1, B2, and B3 were 98.26 ± 0.48%, 96.89 ± 0.99%, and 96.65 ± 1.29%, respectively. The overall difference among the three batches was significant (Kruskal–Wallis *H*_2_ = 6.10, *p* = 0.047), but none of the pairwise comparisons remained significant after Dunn-Bonferroni adjustment (*p* > 0.05; Figure 10A). The corresponding malformation rates for larvae originating from these egg capsules in B1, B2, and B3 were 1.79 ± 0.73%, 2.56 ± 1.19%, and 3.35 ± 1.29%, respectively, with a significant overall difference among the three batches (Kruskal–Wallis: *H*_2_ = 6.00, *p* = 0.0497); Dunn-Bonferroni pairwise comparisons showed that the malformation rate was higher in B3 than in B1 (*p* = 0.044), whereas all other between-group differences were non-significant (all *p* > 0.05; Supplementary Figure S3). By contrast, the natural hatching rates of capsules with spreading deterioration in oviposition batches B1, B2, and B3 were 52.19 ± 5.76%, 47.27 ± 2.30%, and 25.42 ± 16.39%, respectively, and the rate in B3 was lower than those in B1 and B2 (Kruskal–Wallis: *H*_2_ = 13.95, *p* < 0.001; Dunn-Bonferroni, all *p* < 0.05; Figure 10B). The hatching rate was higher in the post-intervention low-temperature incubation group (L, *n* = 7; 65.47 ± 7.26%) than in the natural-condition group (N, *n* = 8; 46.57 ± 7.60%) (Mann–Whitney *U* = 0, *p* < 0.001; Figure 10C). The malformation rate was lower in the L group (5.48 ± 0.78%) than in the N group (12.66 ± 2.58%) (Mann–Whitney *U* = 0, *p* < 0.001; Figure 10D).

## 4. Discussion

### 4.1. Differences Among Breeding Seasons and the Adverse Effects of Mould-like Deterioration in Egg Capsules

Field monitoring across four breeding seasons showed that egg number per egg-capsule pair and the proportion of mould-affected eggs differed among breeding seasons (Figure 2A,B). In 2022–2023, the mean egg number per pair, proportion of mould-affected eggs, and cumulative precipitation were 153.78, 2.05%, and 317.0 mm, respectively. The corresponding values were 150.11, 4.02%, and 390.1 mm in 2023–2024; 118.33, 8.24%, and 255.9 mm in 2024–2025; and 123.33, 7.09%, and 239.2 mm in 2025–2026. Compared with the first two breeding seasons, the latter two had lower egg numbers and higher proportions of mould-affected eggs (Figure 2A,B), alongside lower cumulative precipitation and higher mean monthly winter temperatures (Supplementary Figure S1).

Previous studies have shown that temperature, precipitation, and conditions in oviposition water bodies are associated with egg-stage disease and embryo survival in amphibians [4,37,38,39,40]. The higher proportions of mould-affected eggs observed in 2024–2025 and 2025–2026 coincided with lower precipitation and higher winter air temperatures, suggesting that climatic conditions may contribute to interannual variation. However, the proportion of mould-affected eggs was numerically higher in 2023–2024 than in 2022–2023 despite greater precipitation, whereas it was numerically lower in 2025–2026 than in 2024–2025 despite less precipitation. These patterns suggest that precipitation alone cannot explain the observed interannual variation. Some fungal pathogens can persist outside their hosts and serve as sources of subsequent host exposure [2,41]. Persistent environmental sources of infection may therefore help explain the high proportions of mould-affected eggs in two consecutive breeding seasons, although this possibility requires further investigation. Although field monitoring covered four breeding seasons, sampling was limited to three breeding pools and nine egg-capsule pairs per season. This sampling scope may have limited our ability to detect subtle interannual differences and assess variation among breeding pools. Future studies should increase the number of independent egg-capsule pairs and, where feasible, include additional breeding pools and seasons to characterise finer-scale variation and strengthen the robustness of the findings.

Hypha-like structures and embryonic necrosis occurred together in mould-affected egg capsules (Figure 3H). Similar fungal-associated egg lesions, embryonic mortality, and hatching failure have been reported in other amphibians [4,5,6]. Lesion progression was asynchronous among eggs within the same affected egg capsule: transparent or translucent developing embryos coexisted with grey-white eggs showing structural abnormalities (Figure 3C). This asynchrony indicates that neighbouring embryos may continue to develop after some eggs have developed pronounced mould-like deterioration; a lesion-expansion process therefore exists between the appearance of localised lesions and extensive damage to the egg capsule. During field surveys, we found that some embryos in affected egg capsules completed hatching, although some surviving larvae showed developmental abnormalities (Figure 5A–G). Other egg capsules developed more severe lesions, including extensive grey-white discolouration and collapse of internal egg structures; in some cases, this progressed to mould-like deterioration of all embryos within the capsule (Figure 3A,B,F–H). Associations between early pathogen exposure and post-hatching phenotypic changes have also been reported in other amphibians [42,43]. In those studies, pathogen exposure during embryonic development was accompanied by impaired development, with effects that persisted after hatching as changes in growth, body size, and survival. The larval developmental abnormalities observed here are therefore consistent with sustained pathogen stress and impaired embryonic development during mould-like deterioration.

These findings suggest that the adverse effects of mould-like deterioration in egg capsules cannot be evaluated solely by whether hatching succeeds; the developmental status of surviving larvae should also be considered. This observation supports the use of lesion status to assess natural hatching outcomes and inform subsequent conservation intervention.

### 4.2. Differences in Microbial Communities Among Lesion-Associated Sample Types

Microbial analyses of lesion-associated samples showed that both eukaryotic and bacterial communities differed among sample types. 18S rRNA sequencing showed significant differences in eukaryotic community composition among Stomach, Intestine, and Embryo samples. Ascomycota was the predominant fungal group in Embryo samples, whereas Chytridiomycota and Mucoromycota had higher relative abundances in Intestine samples. Ascomycota includes both widespread saprotrophic fungi and opportunistic fungal pathogens [44]. Chytridiomycota includes known fungal pathogens of amphibians [2,45], and some Mucorales within Mucoromycota are also opportunistic pathogens [46].

Amplicon sequencing can identify differences in community composition and candidate taxa, but it cannot establish the pathogenicity of a particular fungus or bacterium or determine whether microbial community changes preceded or followed lesion development [47,48]. Thus, although Ascomycota was the predominant fungal group in the Embryo samples, whether it is pathogenic to *Hynobius amjiensis* embryos requires further experimental evidence. Chytridiomycota and Mucoromycota had higher relative abundances in the lesion-associated Intestine samples and may serve as candidate taxa for subsequent validation. In the independent 16S dataset, bacterial community composition differed significantly among HF, Embryo, and IC samples, but no single bacterial genus was consistently predominant in lesion-associated samples. Previous studies of amphibians have shown that disease status can be associated with the structure of host-associated bacterial communities and with changes in the relative abundance of particular taxa [49]. These studies emphasise that disease-associated microbial changes should be evaluated at the community level rather than by focusing on a single bacterial taxon [50]. Overall, both 18S and 16S rRNA sequencing showed clear differences in microbial communities among sample groups and identified several candidate taxa associated with lesions, providing a basis for subsequent targeted detection, pathogen isolation, and pathogenicity verification. Because the 18S and 16S datasets were derived from independent biological samples with partially different sample types, they should be interpreted separately rather than as directly complementary microbial profiles. The limited biological replication (n = 3 per group) also constrains the assessment of within-group variability and the robustness of the observed community patterns. Future studies should increase biological replication and adopt a more closely matched sampling design to enable direct comparisons between fungal and bacterial communities.

### 4.3. Natural Hatching Outcomes of Spreading Mould-like Deterioration and Conservation Intervention

Natural hatching outcomes differed markedly between egg capsules with non-spreading and spreading mould-like deterioration. Hatching rates exceeded 96% for capsules with non-spreading deterioration in the first (B1), second (B2), and third (B3) oviposition batches. By contrast, among capsules with spreading deterioration, the hatching rate was lower in B3 (25.42%) than in B1 (52.19%) and B2 (47.27%). These results show that the presence of a small number of mould-affected eggs without further spread does not mean that an entire egg capsule has lost its capacity to hatch, whereas continued spread to neighbouring eggs is associated with a markedly lower hatching rate. Dead eggs or eggs in which development has ceased can provide a substrate for the growth of fungi such as water moulds, increasing the risk of further infection in neighbouring eggs. This phenomenon has been reported in other amphibian eggs [4,5,51]. This pattern is similar to the lesion-expansion process observed in this study. The presence or absence of lesion spread may therefore serve as a phenotypic feature that distinguishes different natural hatching outcomes. Transparent or translucent developing embryos remained in capsules in which deterioration had begun to spread (Figure 3C), and nearly half of the initial eggs in B1 and B2 still hatched naturally. This finding corroborates the observed asynchrony in lesion progression among eggs within the same capsule. It indicates that an intervention window exists between the onset of spread and the cessation of development in all embryos, and that earlier intervention produces better outcomes.

The intervention results showed that the L group had a higher hatching rate and a lower malformation rate than the N group (Figure 10C,D). Michaels’ study of water-mould-like infection in the egg sacs of *Hynobius dunni* emphasised the roles of mechanical removal of lesions and maintenance of egg-sac structure [52]. Amphibian egg jelly also provides a physical barrier and thermal buffering [5,53]. *Hynobius amjiensis* inhabits high-elevation sites; breeding occurs under low-temperature conditions, and breeding pools may freeze in winter [17,20,21]. After eggs become detached from the egg jelly, they may lose the thermal buffering it provides, increasing the risk of abnormal embryonic development or death. The gel refilling used in this study was therefore intended to maintain the position and local structure of the remaining eggs within the treated capsules and to reduce their risk of direct exposure to ambient temperature fluctuations.

Taken together, the combined intervention used in this study was associated with favourable hatching outcomes. After spreading mould-like deterioration developed, embryos that retained developmental capacity subsequently hatched, indicating that a window for conservation intervention remained after the lesions had spread. Together with the natural hatching observations, lesion-spreading status and the continued presence of developing embryos within an egg capsule may help identify capsules that could benefit from intervention.

### 4.4. Conservation Recommendations

We monitored egg-capsule development across multiple breeding seasons, providing guidance for field monitoring and conservation intervention for *Hynobius amjiensis* egg capsules. In field monitoring, priority should be given to recording whether lesions are spreading to neighbouring eggs and whether developing embryos are still present within the egg capsules. For capsules in which no lesion spread is observed and the remaining embryos develop normally, continued observation is appropriate, whereas targeted intervention should be applied to capsules with spreading lesions. In addition, breeding-pool management should be combined with removal of mould-affected material, maintenance of the surrounding vegetation, and monitoring of water level and water quality. In breeding seasons with low precipitation and high temperatures, water-level maintenance, monitoring of breeding-pool fungi, and assessment of the infection risk of mould-affected egg capsules can be strengthened, and targeted protective measures should be implemented according to the monitoring data. Our study provides observational evidence of egg-capsule mould-like deterioration in *Hynobius amjiensis* across four breeding seasons. By linking lesion-spreading status with natural hatching outcomes and conservation intervention, these findings provide a basis for identifying egg capsules that may benefit from timely intervention. The microbial community analyses also identify candidate taxa for subsequent pathogen isolation and pathogenicity verification. Previous studies of cultured fish have highlighted the potential value of species- and life-stage-specific environmental management for animal welfare and conservation practices [54,55]. This lesion-based approach may also inform animal welfare considerations by minimising unnecessary handling of egg capsules with non-spreading lesions while prioritising intervention for capsules with spreading lesions and viable embryos. However, animal welfare outcomes were not directly assessed in this study and require further evaluation.

## 5. Conclusions

This study shows that some cases of mould-like deterioration in *Hynobius amjiensis* egg capsules may represent the decomposition of unfertilised eggs rather than fungal infection. Spreading mould-like deterioration may be associated with fungal infection and may lead to embryonic mortality, hatching failure, and a small number of developmental abnormalities. 18S rRNA sequencing revealed clear differences in fungal communities among affected embryos and related samples; Ascomycota, Chytridiomycota, and Mucoromycota are fungal groups that merit attention in subsequent conservation research. By contrast, 16S rRNA sequencing did not identify a single bacterial genus that was consistently predominant in lesion-associated samples. Statistical analyses of natural hatching outcomes showed that capsules with spreading deterioration had markedly lower hatching rates and higher malformation rates than those with non-spreading deterioration. The proportion of mould-affected eggs may vary among years because of climatic factors or differences in prior pathogen accumulation. Intervention for capsules with spreading deterioration was effective, with a higher hatching rate and a lower malformation rate in the L group than in the N group. These findings can inform conservation efforts for *Hynobius amjiensis* and other amphibians. This study integrates four breeding seasons of field monitoring with lesion phenotyping, microbial community profiling, and conservation intervention. However, the limited biological replication in sequencing and the use of a combined intervention constrain causal interpretation: microbial associations do not establish pathogenicity, and the effects of individual treatment components cannot be determined. Future studies should expand sampling, isolate and experimentally verify candidate pathogens, and evaluate individual intervention components under controlled conditions.

## Data Availability

The data supporting the findings of this study are included in the article and its Supplementary Materials. Additional data are available from the corresponding author [Haiyan Dong], upon reasonable request.

## Supplementary Materials

The following supporting information can be downloaded at: https://www.mdpi.com/article/10.3390/jof12100747/s1.
