# Supplementary material for: Mould-like Deterioration of Anji Salamander (Hynobius amjiensis) Egg Capsules: Adverse Effects, Potential Pathogenic Factors, and Conservation Intervention
Source: J Fungi (Basel). 2026 Oct 5;12(10):747. doi: 10.3390/jof12100747 (PMC13706004; doi:10.3390/jof12100747)
Supplement: Supplementary file 1 [file jof-12-00747-s001.zip › Supplementary_Information.pdf]

Supplementary Information

Supplementary Methods

Supplementary Methods S1. Amplicon sequencing and bioinformatic processing

Total genomic DNA was extracted by Shanghai Majorbio Bio-Pharm Technology Co., Ltd. using its standard protocol for amplicon sequencing. DNA integrity and quality were assessed by electrophoresis on a 1% agarose gel, and samples that passed quality control were used for PCR amplification. Each PCR was performed in a 20 μL reaction mixture containing 10 μL of 2 × ProTaq, 0.8 μL of forward primer (5 μM), 0.8 μL of reverse primer (5 μM) and 10 ng of template DNA, with ddH_2O added to a final volume of 20 μL. The PCR programme began with an initial denaturation at 95 ℃ for 3 min. Each cycle consisted of 95 ℃ for 30 s, the primer-specific annealing temperature for 30 s and 72 ℃ for 45 s. After the specified number of cycles, a final extension was performed at 72 ℃ for 10 min. The 18S rRNA gene was amplified with primers SSU0817F/SSU1196R at an annealing temperature of 55 ℃ for 35 cycles. The 16S rRNA gene was amplified with primers 338F/806R at an annealing temperature of 53 ℃ for 29 cycles. For each sample, PCR was performed in 3 technical replicates, and the replicate amplicons were pooled before assessment by electrophoresis on a 2% agarose gel. Target amplicons were purified using the AxyPrep DNA Gel Extraction Kit (Axygen Biosciences, Union City, CA, USA) and eluted in Tris-HCl buffer. The purified amplicons were quantified using QuantiFluor-ST (Promega, Madison, WI, USA) and pooled in equimolar amounts. Libraries were prepared using the TruSeq DNA Sample Prep Kit and sequenced on an Illumina NextSeq 2000 platform in paired-end mode (2 × 250 bp).

Supplementary Methods S2. Reagent formulations and procedures used in the conservation intervention and observations of embryo hatching

Sterile Ringer's solution was prepared by dissolving 6.50 g NaCl, 0.14 g KCl, 0.12 g $CaCl_2$, 0.20 g $NaHCO_3$ and 0.01 g NaH_2PO_4 in 1000 mL of distilled water. Because autoclaving may alter the pH of $NaHCO_3$ solutions or cause precipitation, the Ringer's solution was sterilised by filtration through a 0.22 μm membrane and dispensed into sterile containers. Before use, the Ringer's solution and the other treatment solutions were pre-cooled to 4-8 ℃. A 0.001% povidone-iodine working solution was prepared by diluting 5% medical-grade povidone-iodine 5000-fold with sterile water. To prepare sterile Ringer's solution containing 2 mg/L vitamin C, vitamin C was dissolved in sterile Ringer's solution, and the solution was filter-sterilised again through a 0.22 μm membrane. To prepare the

0.8% agar gel in sterile Ringer's solution, 0.8 g agar powder was added to 100 mL sterile Ringer's solution and heated until fully dissolved. Under sterile conditions, the gel was cooled to 37 °C or below and used while still fluid.

Supplementary Methods S3. Meteorological data acquisition and correction

Monthly meteorological data for the study period were used to characterise the climatic background of the region containing the breeding site. Raw monthly mean air temperature and monthly precipitation were obtained from records of Anji County Meteorological Station (station ID: 58446). Monthly mean air temperature for the habitat region was estimated by applying the elevation-correction formula described in the main text to the elevation difference between the station and the study area. The corrected temperature was used only to characterise the approximate regional air-temperature background; it does not represent the actual breeding-pool water temperature, which was measured in the field. Monthly precipitation was not corrected for elevation. The breeding season was defined as November of one year to March of the next, and cumulative breeding-season precipitation was calculated by summing monthly precipitation over this period. Monthly meteorological records and aggregation windows for the four breeding seasons spanning November 2022 to March 2026 are provided in Supplementary Table S2. Supplementary Figure S1 compares cumulative precipitation and corrected monthly mean air temperature among the four breeding seasons.

Supplementary Figures

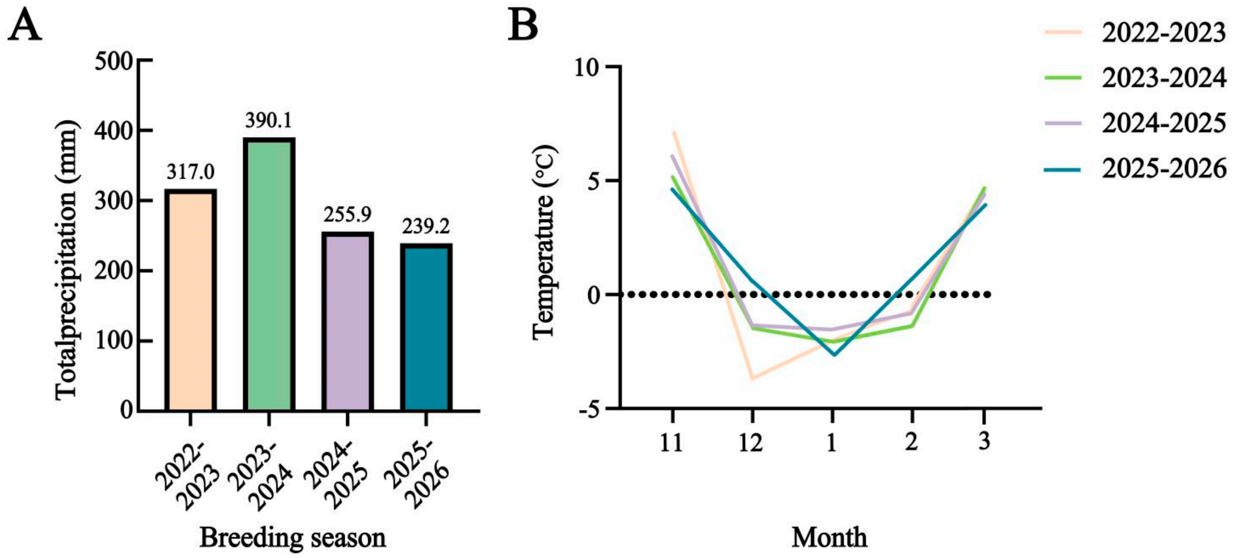

Figure S1. Changes in cumulative precipitation and corrected monthly mean air temperature across four breeding seasons in the Longwangshan habitat region from 2022 to 2026. (A) Cumulative precipitation during the 2022-2023, 2023-2024, 2024-2025 and 2025-2026 breeding seasons. A breeding season was defined as November of one year to March of the next, and numbers above the bars indicate cumulative precipitation (mm). (B) Corrected regional monthly mean air temperature from November to March in each of the four breeding seasons. Coloured lines denote different breeding seasons, and the black dotted horizontal line denotes 0 ℃. Regional air temperature for the habitat was derived from observations at Anji County Meteorological Station and corrected for the elevation difference between the station and the study area. The correction method and parameters are described in the main text and Supplementary Table S2. The corrected temperature characterises the climatic background of the study region and does not represent the actual water temperature in the breeding pool.

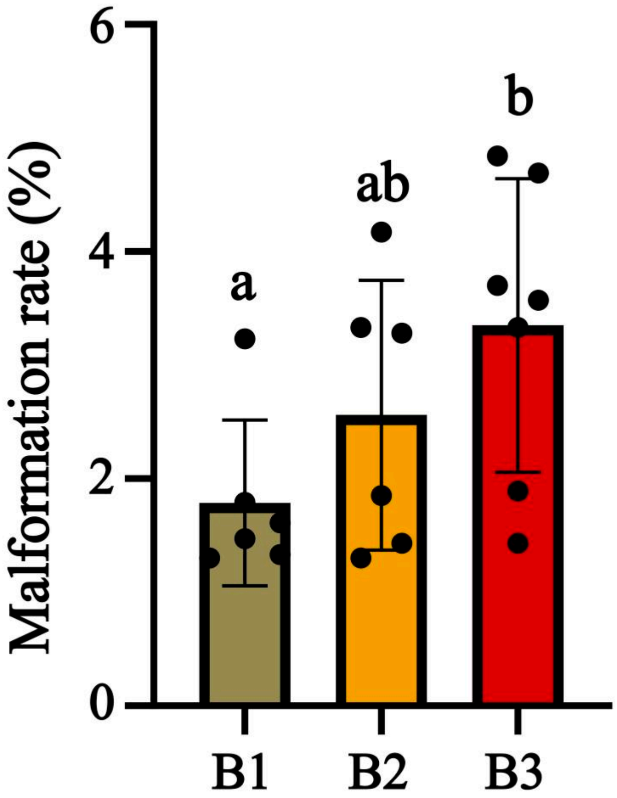

Figure S2. Malformation rate of larvae originating from egg capsules with non-spreading mould-like deterioration in different Anji salamander oviposition batches. Sample sizes for the three oviposition batches B1, B2 and B3 were n = 6, 6 and 7, respectively. Differences among groups were assessed using the Kruskal-Wallis test, followed by Dunn's test with Bonferroni correction for post hoc pairwise comparisons. Different lowercase letters indicate significant differences between groups after correction ($P < 0.05$). Bars and error bars represent mean ± SD, and each point represents 1 independent egg capsule.

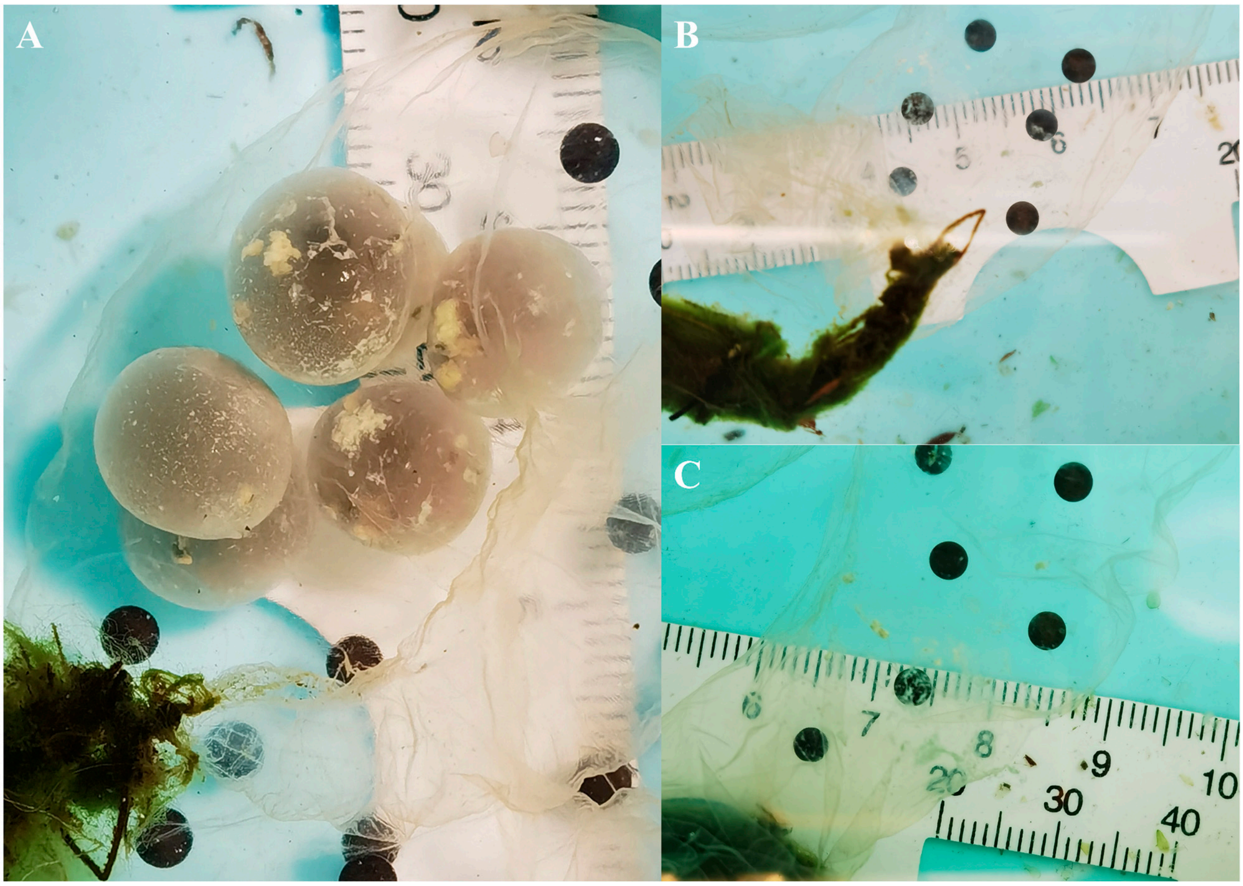

Figure S3. Mould-affected egg capsules before and after the conservation intervention. (A) A mould-affected egg capsule before the intervention, showing multiple greyish-white or mould-affected eggs and visible white hypha-like growth on and around the egg surfaces. (B, C) Egg capsules after the intervention. Localised mould-affected eggs and visible adherent material had been removed, while the remaining egg-jelly structure retained some integrity.

Supplementary Tables S1–S6 are provided as separate Excel files.
